# De Novo Heterozygous *KDM3B* Variants Expand the Mutational Spectrum of Diets-Jongmans Syndrome: Case Series and Literature Review

**DOI:** 10.3390/genes17030294

**Published:** 2026-02-28

**Authors:** Haixia Miao, Ting Zhang, Shuai Chen, Xiaocha Xu, Kexin Fang, Dingwen Wu, Yi Zhang, Xinwen Huang

**Affiliations:** Department of Genetics and Metabolism, Children’s Hospital, Zhejiang University School of Medicine, National Clinical Research Center for Children and Adolescents’ Health and Disease, Hangzhou 310051, China

**Keywords:** *KDM3B* gene, Diets-Jongmans syndrome, growth retardation, whole-exome sequencing

## Abstract

Background: Pathogenic variants in *KDM3B* have been implicated as the cause of Diets-Jongmans syndrome (DIJOS), an autosomal-dominant disorder characterized by growth retardation, intellectual disability, facial dysmorphism and autism-spectrum disorder. However, only a limited number of cases have been reported. Methods: The general characteristics of four patients were recorded, including clinical features, child development, neuropsychological assessment and therapeutic interventions. Whole exome sequencing (WES) was performed for potential genetic causes and interpretation of variants was performed in accordance with ACMG guidelines. Results: All patients carried de novo variants in the *KDM3B* gene, namely, c.2832-3C>G, c.1188del p.(Glu397Argfs*21), c.4580T>C p.(Leu1527Pro), and c.3220dup p.(Glu1074Glyfs*48). Unlike other patients with DIJOS who presented with growth retardation, mild to moderate intellectual developmental disorder and facial dysmorphism, our patients mainly presented with growth retardation, while their neurodevelopment was either normal or mildly impaired. In addition, our patients received primarily supportive care. One patient treated with recombinant human growth hormone (rhGH) showed improvement in growth. Conclusions: Our results broaden the mutational spectrum of *KDM3B*-related disorder and highlight the inter-patient variability of the clinical phenotype. For the first time, we demonstrate that rhGH therapy can partially promote growth, providing novel evidence for genetic counseling.

## 1. Introduction

KDM3B is an enzyme that takes part in histone demethylation and epigenetic regulation, which belongs to the KDM3 family [1]. KDM3B simultaneously possesses lysine demethylase (KDM) and arginine demethylase (RDM) activities, making it a bifunctional demethylase. The enzyme specifically removes one or two methyl groups from H3K9me2 and H4R3me2 marks, and this dual functionality contributes to the development of hematopoietic stem cells (HSCs) and progenitor cells [2].

In 2019, Diets et al. reported heterozygous *KDM3B* variants in 17 unrelated individuals with developmental delay and formally designated this autosomal-dominant conditions as Diets-Jongmans syndrome (DIJOS; MIM#618846). The disorder is characterized by developmental delay, mild-to-moderate intellectual disability and facial dysmorphism, which includes macrostomia, pointed chin, long ears, broad nasal bridge and thin upper vermilion. Approximately two-thirds of patients exhibit short stature. Additional variable features encompass autism-spectrum disorder, attention-deficit hyperactivity disorder (ADHD), and congenital diaphragmatic, umbilical or inguinal hernias [3].

However, Somin Jo et al. [4] reported a male patient with a de novo frameshift variant in the *KDM3B* gene, who presented with short stature, slow weight gain and mild motor delays. In contrast to other DIJOS patients, this patient lacked obvious signs of neurodevelopmental delay. In our study, all four patients harboring de novo *KDM3B* variants exhibited growth retardation, while their neurodevelopment was either normal or only mildly impaired. These findings suggest that DIJOS exhibits phenotypic heterogeneity, and growth retardation seems to be a highly consistent phenotype in these patients.

Using whole-exome sequencing, we identified four patients with DJIOS harboring de novo variants in the *KDM3B* gene, all of which are reported for the first time. Distinct from previously described cases, the four patients in this study exhibited growth retardation as the predominant clinical feature, with only one patient displaying mild neurodevelopmental delay. Furthermore, our study provides the first evidence that recombinant human growth hormone (rhGH) therapy can effectively promote growth in patients with DJIOS, thereby establishing an important foundation for precision clinical management and informed genetic counseling for this syndrome.

## 2. Materials and Methods

### 2.1. Patients

This study included four unrelated patients with DIJOS, who were recruited at the Children’s Hospital, Zhejiang University School of Medicine between 2023 and 2024. Comprehensive clinical data of each proband were retrospectively collected, including clinical features, child development, neuropsychological assessment results and therapeutic interventions. Prior to genetic testing, written informed consent was obtained from the guardians for genetic analyses. Additionally, this study was approved by the Ethical Committee of the Children’s Hospital Zhejiang University School of Medicine, with the approval number 2020-IRBAL-035.

### 2.2. DNA Isolation

Peripheral blood samples (2 mL) were collected from all patients and their parents for genetic analysis. Genomic DNA was extracted using the MagBio Blood Spots Genomic DNA Purification Kit (Bioer Tech., Hangzhou, China) following the manufacturer’s standard protocol. DNA concentration was determined using the Qubit dsDNA HS Assay Kit (Yeasen, Shanghai, China); only samples yielding ≥ 200 ng total DNA, a concentration > 20 ng/μL and an A260/280 ratio of 1.7–2.0 were accepted for following analysis.

### 2.3. Whole-Exome Sequencing

Exome capture on all probands and their parents was performed using the KAPA HyperExome V2 Probes kit (Roche Molecular Systems, Inc., Pleasanton, CA, USA), which covers all coding exons and flanking intronic regions. Paired-end sequencing (150 bp) was performed on the MGI DNBSEQ-T7 platform (MGI Tech., Beijing, China) to achieve a mean sequencing coverage of over 120×, with more than 98.5% of the target bases covered at least 20×. High-quality, QC-passed reads were aligned to the GRCh37/hg19 reference genome via BWA software (v2.2.1). Single-nucleotide variants (SNVs) and small insertions/deletions (indels) were called with GATK v4.0. Identified variants were annotated and classified using VEP (v113) and ANNOVAR (v20250302), and population allele frequencies were extracted from gnomAD (v2.1.1; https://gnomad.broadinstitute.org; accessed on 1 August 2025), and dbSNP (http://www.ncbi.nlm.nih.gov/snp/, accessed on 19 June 2025). Sequence conservation, amino acid substitutions and structural context were annotated using OMIM (http://www.ncbi.nlm.nih.gov/omim, accessed on 20 November 2025), HGMD (http://www.hgmd.org/, accessed on 26 September 2025), and ClinVar (http://www.ncbi.nlm.nih.gov/clinvar/, accessed on 1 August 2025).

Variant filtering was performed by excluding common variants with a minor allele frequency (MAF) > 2% or an allele number (AN) > 2000 in gnomAD (v2.1.1; https://gnomad.broadinstitute.org; accessed on 1 August 2025), and an in-house database. High-confidence benign variants in ClinVar (https://www.ncbi.nlm.nih.gov/clinvar; accessed on 1 August 2025) were removed, unless they had been previously classified as pathogenic/likely pathogenic, variants of uncertain significance, or variants with conflicting interpretations. Variants absent from HGMD (http://www.hgmd.org/, accessed on 26 September 2025) and with a SpliceAI (https://spliceailookup.broadinstitute.org, accessed on 19 July 2025) delta score < 0.5 were further filtered out. A prioritization framework was applied to rank remaining variants based on the following criteria: phenotypic congruence, inheritance patterns (prioritizing de novo (AD) and homozygous (AR/XL), compound heterozygous (AR), autosomal recessive (AR), and X-linked recessive (XL) patterns), pathogenicity evidence (integrated from ClinVar, HGMD, REVEL, SIFT, and SpliceAI), and sequencing quality metrics (variant allele frequency (VAF) and read depth). Candidate variants were determined through comprehensive evaluation of the aforementioned multi-dimensional factors.

### 2.4. Sanger Sequencing

Candidate variants selected based on filtering/prioritization criteria were subsequently validated by Sanger sequencing. PCR was carried out in a 25 µL volume containing 50 ng genomic DNA, 1 µL each of 10 pmol forward and reverse primers, 12.5 µL 2 × Phanta Max Master Mix (Vazyme, Nanjing, China), and nuclease-free water. The thermal cycling protocol was as follows: 95 °C for 2 min; 36 cycles of 95 °C for 30 s, 58 °C for 30 s, 72 °C for 1 min; final extension 72 °C for 2 min. Amplicons were resolved on 1.5% agarose gels and subsequently analyzed by capillary electrophoresis on an ABI 3730xl (Applied Biosystems, Foster City, CA, USA) DNA Analyzer and sequencing products were analyzed using DNA StarMegalign software (version 3.3.8; DNASTAR, Inc., Madison, WI, USA).

### 2.5. Literature Search Strategy

A systematic literature review was conducted using CNKI (https://www.cnki.net), VIP (https://www.cqvip.com), Wanfang Data Knowledge Service Platform (https://www.wanfangdata.com.cn) and PubMed (https://pubmed.ncbi.nlm.nih.gov) from database inception to November 2025 with the terms “*KDM3B* gene” and “Diets-Jongmans syndrome”.

### 2.6. Three-Dimensional Modeling of the KDM3B Protein Mutation Site

The three-dimensional structure of lysine-specific demethylase 3B (KDM3B, Uniprot accession: Q7LBC6.1) was generated using the SWISS-MODEL homology modeling server. The target sequence was retrieved from the Uniprot and modeled against the AlphaFold-derived template Q7LBC6.1.A (100% sequence identity) to ensure maximum modeling accuracy. Model 01, selected for downstream analyses, was generated using the integrated algorithm in SWISS-MODEL and exhibited the highest overall quality. Quality assessment confirmed a monomeric architecture and a Global Model Quality Estimation (GMQE) score of 0.58. Template–target alignment revealed complete sequence identity, which further validated the reliability of the constructed 3D model.

## 3. Results

### 3.1. Clinical Features

**Figure 1:**
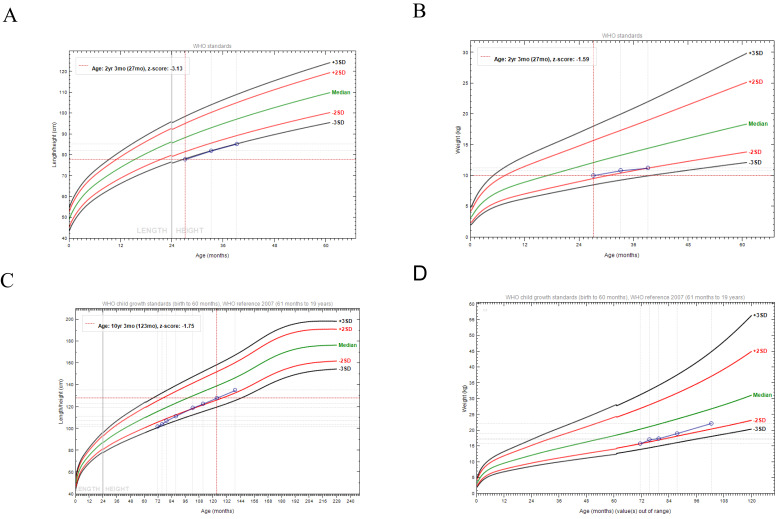

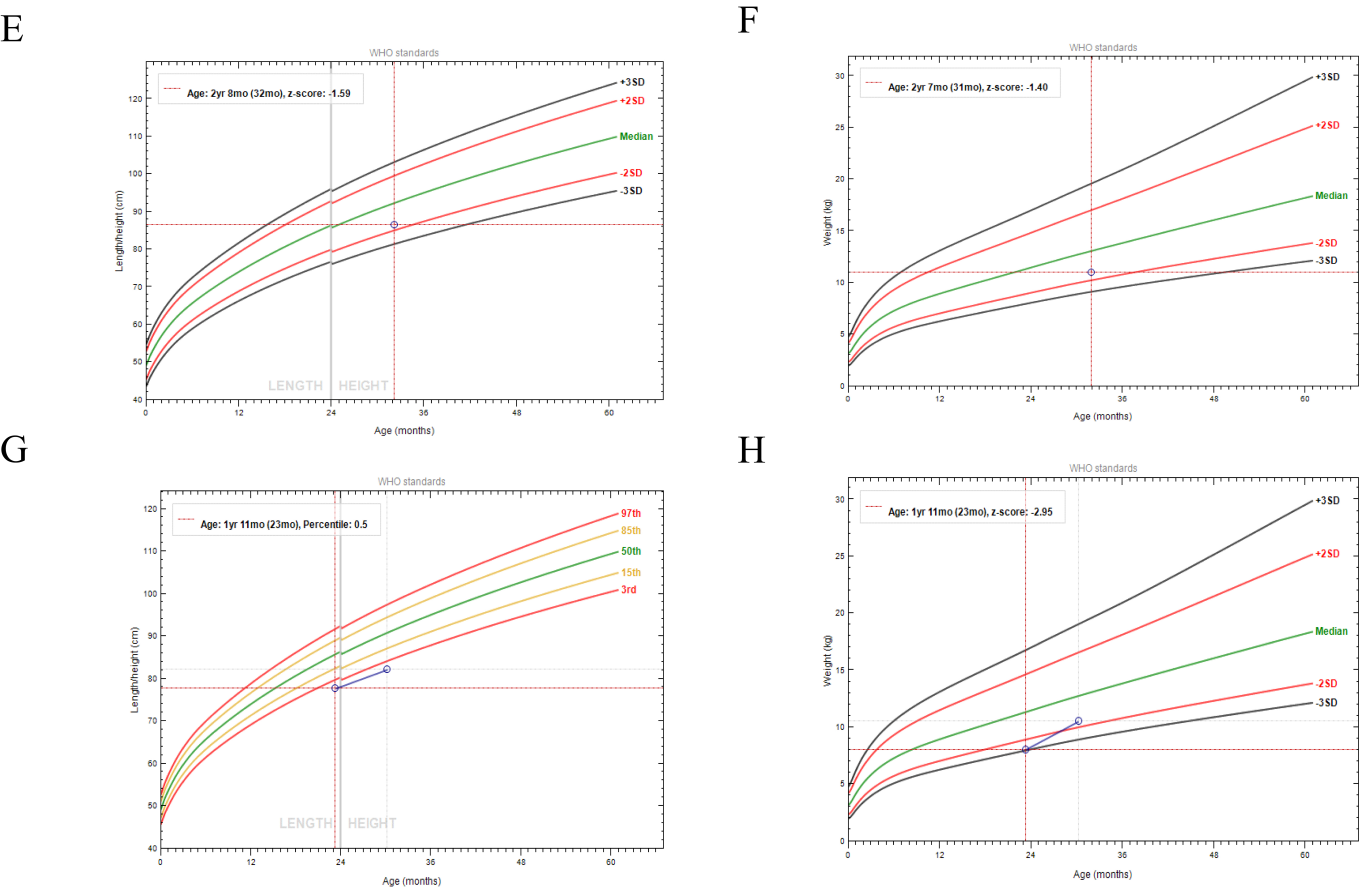
Height and weight growth curves of the four patients. Circles: data points for height and weight at each measurement. (**A**) Height curve of P1; (**B**) Weight curve of P1; (**C**) Height curve of P2; (**D**) Weight curve of P2; (**E**) Height curve of P3; (**F**) Weight Curve of P3; (**G**) Height curve of P4; (**H**) Weight curve of P4.

The patients were recruited from four unrelated families, and none of their parents were consanguineous. All patients were born at term; one had low birth weight and the remaining three had normal birth weight. The average age of the four patients was 4 years and 3 months. Although birth length was normal (48–50 cm) in all patients, feeding difficulties were present in all of them, followed by the growth retardation. Their height and weight ranged from −2 SD to −3 SD (Figure 1/Table 1). The Gesell Developmental Schedules for P1 showed scores of 82 (adaptive), 71 (gross motor), 83 (fine motor), 90 (language), and 74 (personal-social). P2 achieved an IQ score of 117 on Raven’s Progressive Matrices; attention function testing revealed a score of 36 with an error rate of 12.5%; SNAP-IV scores of 21-6-0 (inattention-hyperactivity/impulsivity-opposition) confirmed the diagnosis of ADHD, predominantly inattentive type. Although P3 and P4 lacked formal neurodevelopmental assessment, no obvious neurodevelopmental abnormalities were observed in clinical examinations. Additionally, P2 received recombinant rhGH therapy starting from May 2019, with an initial dose of 1.5 U/day which was gradually increased to 5.9 U/day. The height velocity was approximately 6.5 cm/year, with height increasing from 94.3 cm to 135 cm (May 2019 to August 2025). Bone age progressed from delayed to within the normal range. During the treatment course, the patient was followed up every 3 months at the endocrine outpatient clinic for monitoring of serum insulin, IGF-1 and binding proteins, thyroid function and blood glucose levels, all of which remained within normal ranges.

### 3.2. KDM3B Variants

**Figure 2:**
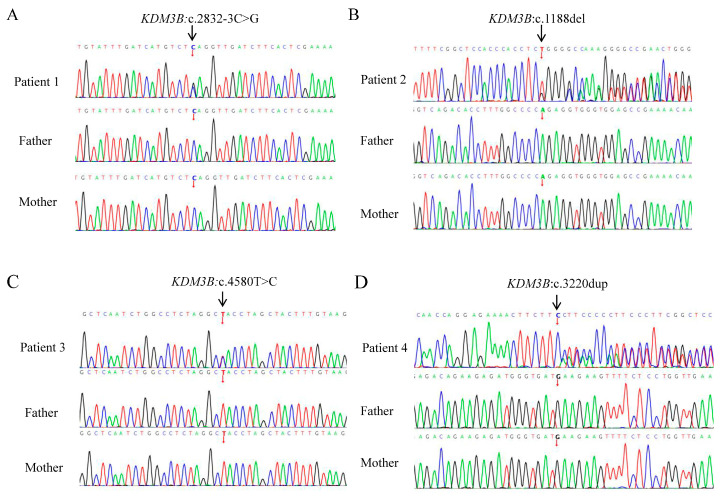
Sanger sequencing chromatograms of probands and parents confirming the de novo origin of *KDM3B* variants (NM_016604.4). (**A**) Patient 1: c.2832-3C>G; (**B**) Patient 2: c.1188del p.(Glu397Argfs*21); (**C**) Patient 3: c.4580T>C p.(Leu1527Pro); (**D**) Patient 4: c.3220dup p.(Glu1074Glyfs*48). Both black and red arrows indicate the variant positions. The chromatograms demonstrate that all variants are absent in all parents.

We identified four distinct de novo heterozygous variants in the *KDM3B* gene (NM_016604.4), namely, c.2832-3C>G, c.1188del p.(Glu397Argfs*21), c.4580T>C p.(Leu1527Pro), and c.3220dup p.(Glu1074Glyfs*48); no other variants remained that could potentially explain the observed phenotype. These variants have not been previously reported in the literature or public databases. In addition, selected variants were validated by Sanger sequencing, and their de novo origin was confirmed (Figure 2A–D). Pathogenicity classification was performed according to the ACMG guidelines [5] (Table 2).

### 3.3. Literature Review

**Figure 3:**
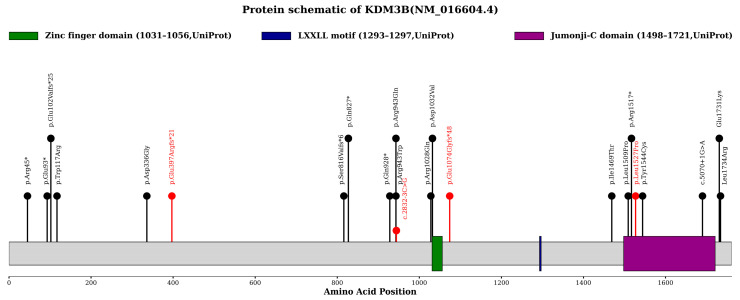
A schematic diagram of KDM3B, including the zinc-finger domain, LXLL motif, and Jumonji-C (JmjC) domain, showing the positions of the reported variants based on their locations at the protein level. Black lollipops represent previously reported cases, while red lollipops represent the cases identified in this study. *: stop codon.

Six studies have reported DIJOS caused by *KDM3B* variants, involving 23 patients [3,4,6,7,8,9] (Table 3). Combined with our study, a total of 27 patients with DIJOS have been described to date. Among these patients, 23 distinct *KDM3B* variants have been identified, including 12 missense variants, 5 nonsense variants, 4 frameshift variants and 2 splice-site variants (Figure 3). Four of these 23 variants were recurrent: c.277G>T p.(Glu93*), c.1007A>G p.(Asp336Gly), c.2828G>A p.(Arg943Gln), and c.5191G>A p.(Glu1731Lys), each detected in two unrelated patients. In terms of variant origin, the vast majority were sporadic de novo variants, with only a minority being familial.

### 3.4. Three-Dimensional Structural Analysis of KDM3B Protein Variant and Secondary Structure Analysis of the JmjC Domain

**Figure 4:**
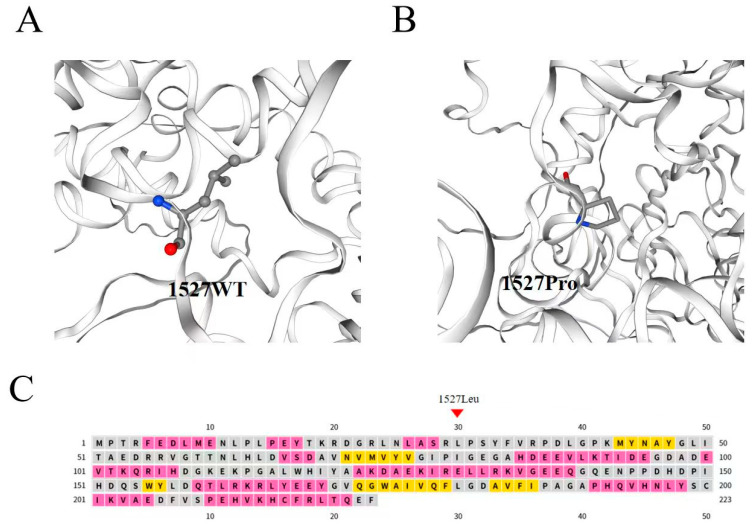
Three-dimensional structural prediction of the KDM3B Leu1527Pro variant and secondary structure analysis of the JmjC Domain (1497–1521). (**A**): Wild-type KDM3B protein; Blue spheres: nitrogen atoms; Red spheres: oxygen atoms; Grey spheres: carbon atoms. (**B**): Mutant KDM3B protein p.(Leu1527Pro); (**C**): The secondary structure prediction for the Jumonji C domain (1497–1521).

The c.4580T>C variant results in the substitution of leucine with proline at position 1527 (p.Leu1527Pro) of the KDM3B protein. To investigate its potential structural impact, we performed in silico three-dimensional structural prediction and pairwise alignment of wild-type (WT) and mutant KDM3B using the SWISS-MODEL server (https://swissmodel.expasy.org/). We selected Q7LBC6.1.A (AlphaFold DB model of KDM3B_HUMAN) as the template, which shared 100% sequence identity with the target protein and a Global Model Quality Estimation (GMQE) score of 0.58. Secondary structure prediction via PSIPRED confirmed that residue 1527 is located in the flanking coil/loop region of the predicted α-helical segment (residues 1523–1525) within the JmjC domain, rather than within the α-helix core itself (Figure 4C). Physicochemically, leucine is a hydrophobic amino acid with a flexible branched aliphatic side chain that stabilizes local protein folds through hydrophobic interactions in coil/loop regions. In contrast, proline is a rigid imino acid with a cyclic side chain covalently linked to the peptide backbone, introducing a sharp backbone kink that disrupts the flexibility of adjacent segments, even in non-helical regions.

In the WT model (Figure 4A), Leu1527 resides in a loose coil/loop adjacent to the α-helix (1523–1525), with its side chain oriented toward the solvent-exposed surface, thereby maintaining local conformational flexibility. In the mutant model (Figure 4B), the Leu1527Pro substitution induces a distinct backbone kink at this position, restricting the conformational freedom of the adjacent peptide chain and subtly altering the spatial arrangement of the flanking α-helix, potentially compromising helical continuity.

## 4. Discussion

Heterozygous pathogenic or likely pathogenic variants in *KDM3B* are associated with DIJOS. To date, 23 patients with *KDM3B* variants and presenting with DIJOS-related phenotypes have been described in the literature [3,4,6,7,8,9]. The core phenotypes include mild-to-moderate intellectual disability or developmental delay, predominantly affecting motor and language function, facial dysmorphism (long ears, broad nasal tip, low columella, thin lips and wide mouth), growth retardation, and neonatal feeding difficulties. Additional features include neurological comorbidities (ADHD, epilepsy and autism spectrum disorder), neonatal hypotonia, respiratory insufficiency, disorders of sex development, and sensorineural hearing loss. Phenotypic expression is highly variable, and pathogenic variants may be inherited from unaffected or mildly affected parents (Table 3).

In this study, we identified four patients with DIJOS caused by de novo *KDM3B* mutations. The main clinical phenotype was growth retardation, with either mild neurodevelopment impairment or normal neurodevelopment. However, previously reported DIJOS cases commonly presented with intellectual disability and facial dysmorphisms in addition to growth retardation [6,7]. Further analysis revealed that the five patients described by Diets et al. who harbored de novo nonsense (loss-of-function, LOF) mutations exhibited no or only mild intellectual/developmental delay, whereas the likelihood of mild-to-moderate intellectual disability was higher among the twelve patients with de novo missense mutations. This finding suggests that LOF variants may confer a relatively milder neurodevelopmental phenotype [3,4]. Jo et al. reported a patient carrying a *KDM3B* frameshift variant who presented primarily with growth delay and no evidence of neurodevelopmental impairment [4]. Among the four mutations identified in this study, three were LOF variants, a pattern largely consistent with the aforementioned hypothesis. However, due to limited sample size and the young age of the patients at the time of assessment, long-term, large-scale studies are needed to further elaborate on the potential relationship between truncating variants and neurodevelopmental outcomes.

*KDM3B* is a protein-coding gene located on chromosome 5q31.2. It consists of 24 exons and encodes histone lysine demethylase 3B [10]. KDM3B is abundantly expressed in multiple murine testicular cell types. Male mice with germline deletion of *Kdm3B* exhibited reduced litter sizes when mated with wild-type females, along with decreased sperm reserves and markedly impaired motility in the cauda epididymis, underscoring the indispensable role of KDM3B in normal spermatogenesis and reproductive behavior [11]. Studies have shown that KDM3B orchestrates biomechanically induced osteogenic differentiation of adipose-derived stem cells [12]. Studies using heterozygous *Kdm3B*-knockout (*Kdm3B*^+^/^−^) mice have shown that diminished H3K9 demethylase activity impairs cerebellar memory consolidation, indicating that KDM3B plays an essential role in motor learning [13].

Notably, postnatal somatic growth restriction in *Kdm3b-null* mice is closely associated with reduced levels of insulin-like growth factor binding protein-3 (IGFBP-3) in the kidney and blood, as well as reduced serum insulin-like growth factor-1 (IGF-1). These low IGF-1 levels indicate impairment of the growth hormone axis, providing a plausible explanation for the short stature observed in patients carrying *KDM3B* variants [14,15]. Furthermore, it is hypothesized by Faundes et al. [16] that variants in genes promoting transcriptional activity are associated with growth retardation, whereas overgrowth is caused by variants in transcriptional repressors. As H3K9 methylation is widely recognized as a repressive epigenetic mark, demethylation of H3K9 by KDM3B is predicted to result in transcriptional activation, which might explain the growth retardation observed in affected patients. Additionally, research on family genes provides valuable evidence. For example, like KDM3B, KDM5C also belongs to the JmjC lysine demethylase family. Experiments in *KDM5C*-knockout mice showed that loss of *KDM5C* severely suppresses in vitro chondrogenic differentiation of mesenchymal stem cells and reduces mRNA levels of chondrogenic factor, resulting in short stature. These parallel findings reinforce the conclusion that *KDM3B* variants can cause short stature [17].

KDM3B contains three functional domains: an N-terminal zinc finger (ZF)-like domain that mediates DNA binding, an LXXLL motif that facilitates interaction with nuclear receptors, and a C-terminal JmjC domain that possesses catalytic activity [10]. To date, 23 *KDM3B* variants have been reported, including those identified in this study and previously published cases. Of these, 11 are LoF variants-comprising 5 nonsense, 4 frameshift, and 2 splice-site variants—while the remaining 12 are missense variants. In this study, we identified two frameshift variants: c.1188del p.(Glu397Argfs*21) and c.3220dup p.(Glu1074Glyfs*48). These variants introduce premature termination codons at residues 418 and 1122, respectively, and are predicted to trigger nonsense-mediated decay (NMD) or, if translated, to produce C-terminally truncated proteins lacking all three functional domains. The c.2832-3C>G variant is located in an intronic region. In silico splicing prediction tools, including SpliceAI and dbscSNV, predict that this variant disrupts canonical mRNA splicing and likely impairs protein function. The c.4580T>C p.(Leu1527Pro) missense variant is located within the JmjC domain and is predicted to impair demethylase activity. In silico three-dimensional structural modeling (SWISS-MODEL) predicts that the substitution of leucine 1527 with proline would introduce a local backbone kink, which may induce subtle torsion and refolding of the adjacent peptide chain. This predicted conformational change could alter the spatial arrangement of the flanking α-helical segment (residues 1523–1525, as predicted by PSIPRED), ultimately perturbing the local tertiary architecture of the KDM3B JmjC domain (Figure 4). Notably, all structural interpretations presented here are hypothetical, being derived from computational predictions. Experimental validation is required to confirm the predicted local conformational changes and their functional implications for KDM3B enzymatic activity.

Currently, there is no curative therapy for DIJOS caused by *KDM3B* variants. Management is supportive and requires a multidisciplinary approach. Children with developmental delay should be referred early to rehabilitation services. Individualized programs addressing cognitive and motor deficits should be initiated promptly to optimize adaptive function and independence. ADHD should be managed with behavioral interventions and pharmacotherapy as indicated. Short stature was observed in nearly half of the patients with DIJOS [3]. P2 received rhGH therapy from May 2019 to August 2025; his height had increased from 94.3 cm to 135 cm, moving from below −3 SD to between −1 SD and −2 SD. The annual growth velocity has remained within the normal reference range, indicating a measurable therapeutic benefit. However, given that KDM3B has been linked to myelodysplastic syndrome and acute myeloid leukemia [18,19], the potential oncogenic risk of rhGH must be carefully weighed. Larger cohorts and longer follow-up are essential to establish the efficacy and long-term safety of rhGH in patients with DIJOS.

We present four patients with de novo *KDM3B* variants. Unlike other previously reported patients with DIJOS, the patients in the present study exhibited normal neurodevelopment or only subtle signs of neurodevelopmental impairment, highlighting that growth retardation could be a highly consistent phenotype in these patients. Moreover, one patient in this study showed improved growth following rhGH therapy, suggesting that rhGH treatment may confer a beneficial effect on growth in individuals with DIJOS.

## Figures and Tables

**Table 1:** General characteristics of four patients.

Information	Patient 1	Patient 2	Patient 3	Patient 4
Gender	F	M	F	F
Gestational age (W)	39	40	38	40
Birth weight (kg)	2.4	3.5	2.65	2.8
Age at presentation	2 Y 3 M	10 Y 4 M	2 Y 8 M	1 Y 11 M
Height (cm)	77.8	126.9	85	76.9
Weight (kg)	10	26	11	8
Height SDS	−3 SD	−3 SD	−2 SD	−3 SD
Weight SDS	−2 SD	−3 SD	−2 SD	−3 SD

W: Week, Y: Year, M: Month.

**Table 2:** *KDM3B* variants interpretation of the four patients.

Patient	Position (hg19)	Nucleotide Change (NM_016604.4)	AAchange (NP_057688.3)	GnomAD	SIFT	PolyPhen-2	Mutation Taster	SpliceAI	DbscSNV	ACMG Classification
P1	chr5:137733864	c.2832-3C>G	/	0	NA	NA	NA	Y	Y	PS2 + PM2
P2	chr5:137722118	c.1188del	p.(Glu397Argfs*21)	0	NA	NA	NA	NA	NA	PVS1 + PS2 + PM2
P3	chr5:137762955	c.4580T>C	p.(Leu1527Pro)	0	deleterious	probably damaging	probably damaging	NA	NA	PS2 + PM2
P4	chr5:137750841	c.3220dup	p.(Glu1074Glyfs*48)	0	NA	NA	NA	NA	NA	PVS1 + PS2 + PM2

NA: not applicable. *: stop codon

**Table 3:** Clinical characteristics of the current patients and previously reported DIJOS individuals.

	This Study	Diets IJ [3]	Jo S [4]	Tabaku M [6]	Zhao X [7]	Li [8]	Benslimane Z [9]	Total Individuals (%)
**Development**								
Intellectual disability or developmental delay	1/4	16/17	0/1	1/1	2/2	1/1	1/1	22/27 (81.5%)
Motor delay	0/4	17/17	1/1	1/1	0/2	0/1	0/1	19/27 (70.4%)
**Growth**								
Height (−2 sd)	4/4	8/17	1/1	1/1	0/2	0/1	1/1	15/27 (55.6%)
Weight (−2 sd)	4/4	11/17	1/1	0/1	0/2	0/1	0/1	16/27 (59.3%)
**Neurological**								
ADHD	1/4	4/17	0/1	0/1	0/2	0/1	0/1	5/27 (18.5%)
Autism spectrum disorder	0/4	3/17	0/1	1/1	0/2	0/1	0/1	4/27 (14.8%)
Epilepsy	0/4	3/17	0/1	0/1	0/2	0/1	0/1	3/27 (11.1%)
Childhood hypotonia	0/4	5/17	0/1	0/1	0/2	0/1	1/1	6/27 (22.2%)
**Congenital Anomalies**								
Facial deformity	0/4	10/17	1/1	1/1	0/2	0/1	1/1	13/27 (48.1%)
Umbilical hernia	0/4	2/17	0/1	0/1	1/2	0/1	0/1	3/27 (11.1%)
Inguinal hernia	0/4	2/17	0/1	0/1	0/2	0/1	0/1	2/27 (7.4%)
Cryptorchidism	0/4	1/17	0/1	0/1	0/2	0/1	0/1	1/27 (3.7%)
Congenital hypothyroidism	0/4	1/17	0/1	0/1	0/2	0/1	1/1	2/27 (7.4%)
**Other**								
Neonatal feeding difficulties	4/4	9/17	0/1	1/1	0/2	0/1	0/1	14/27 (51.9%)
Respiratory system diseases	0/4	0/17	0/1	0/1	2/2	0/1	1/1	3/27 (11.1%)
Hearing loss	0/4	4/17	0/1	0/1	0/2	0/1	0/1	4/27 (14.8%)
Congenital heart disease	0/4	0/17	0/1	0/1	1/2	0/1	1/1	2/27 (7.4%)
Neutropenia	0/4	0/17	0/1	0/1	1/2	0/1	0/1	1/27 (3.7%)
Constipation	0/4	0/17	0/1	0/1	1/2	0/1	0/1	1/27 (3.7%)
Cardiomyopathy	0/4	0/17	0/1	0/1	1/2	0/1	0/1	1/27 (3.7%)

## Data Availability

The data generated in this study can be found within the article. Raw data are available from the corresponding author on request.
